# Poor maternal nutrition inhibits muscle development in ovine offspring

**DOI:** 10.1186/2049-1891-5-43

**Published:** 2014-09-05

**Authors:** Sarah A Reed, Joseline S Raja, Maria L Hoffman, Steven A Zinn, Kristen E Govoni

**Affiliations:** 1 Department of Animal Science, University of Connecticut, Storrs, CT 06269, USA

**Keywords:** Growth, Maternal nutrition, Muscle development, Sheep

## Abstract

**Background:**

Maternal over and restricted nutrition has negative consequences on the muscle of offspring by reducing muscle fiber number and altering regulators of muscle growth. To determine if over and restricted maternal nutrition affected muscle growth and gene and protein expression in offspring, 36 pregnant ewes were fed 60%, 100% or 140% of National Research Council requirements from d 31 ± 1.3 of gestation until parturition. Lambs from control-fed (CON), restricted-fed (RES) or over-fed (OVER) ewes were necropsied within 1 d of birth (n = 18) or maintained on a control diet for 3 mo (n = 15). Semitendinosus muscle was collected for immunohistochemistry, and protein and gene expression analysis.

**Results:**

Compared with CON, muscle fiber cross-sectional area (CSA) increased in RES (58%) and OVER (47%) lambs at 1 d of age (*P* < 0.01); however at 3 mo, CSA decreased 15% and 17% compared with CON, respectively (*P* < 0.01). Compared with CON, muscle lipid content was increased in OVER (212.4%) and RES (92.5%) at d 1 (*P* < 0.0001). Muscle lipid content was increased 36.1% in OVER and decreased 23.6% in RES compared with CON at 3 mo (*P* < 0.0001). At d 1, *myostatin* mRNA abundance in whole muscle tended to be greater in OVER (*P* = 0.07) than CON. *Follistatin* mRNA abundance increased in OVER (*P* = 0.04) and tended to increase in RES (*P* = 0.06) compared with CON at d 1. However, there was no difference in myostatin or follistatin protein expression (*P* > 0.3). Phosphorylated Akt (ser473) was increased in RES at 3 mo compared with CON (*P* = 0.006).

**Conclusions:**

In conclusion, maternal over and restricted nutrient intake alters muscle lipid content and growth of offspring, possibly through altered gene and protein expression.

## Background

Poor maternal nutrition can be defined as the overall nutritional excess or restriction during gestation as well as excess or lack of specific nutrient classes, such as proteins or minerals. Restricted- or over-feeding during gestation has immediate and long-term impacts on growth and development of offspring [1-4]. The type and extent of phenotypic alterations vary depending on the duration, type, and form of inappropriate maternal nutrition. Maternal over or restricted nutrition during gestation can affect pre- and post-natal muscle growth, altering skeletal muscle fiber number, intramuscular adiposity, and fiber type composition [5-7]. The fetal period is crucial to muscle development. In sheep, muscle fiber formation begins around d 20 and is completed between d 80 and 125 of gestation [8,9], after which fiber number is fixed. Postnatal muscle growth occurs through hypertrophy with increases in protein accretion and contributions from muscle satellite cells. Satellite cell activity can be evaluated by the expression of Pax7 and the myogenic regulatory factors (MRF) MyoD, Myf5, myogenin and MRF4 as markers of specific stages in myogenesis [10,11]. A previous study demonstrated that muscle from 75-d old fetuses from ewes fed an obesogenic diet exhibited reduced gene expression of *MyoD* and *mgn* compared with fetuses from control ewes [12].

Although the various phenotypic effects of maternal restricted- and over-nutrition on offspring growth and development are known, the mechanisms through which poor maternal nutrition alters animal body composition, particularly muscle development, are not well understood. We hypothesized that over and restricted nutrition (total nutrient intake) in gestating ewes would negatively affect muscle growth in the offspring by reducing muscle fiber size, and altering gene and protein expression via the Akt signaling pathway. The objective of this study was to determine the effects of maternal over or restricted nutrition during gestation on muscle development in the offspring.

## Methods

### Animals

All animal experiments were reviewed and approved by the University of Connecticut Institutional Animal Care and Use Committee.

Thirty-six multiparous ewes (25 Dorsets, 7 Shropshires, and 4 Southdowns) were selected from the University of Connecticut sheep flock and bred within breed by live cover to 1 of 5 different rams (3 Dorsets, 1 Southdown and 1 Shropshire) following estrous synchronization with progesterone controlled intravaginal drug release devices (Pfizer Animal Health, New York, NY) and Lutalyse (Pfizer Animal Health [13,14]). Date of breeding was considered the day that ewes were marked by the ram. At approximately d 30 of gestation, ewes were moved into individual pens. Pregnancy was confirmed using ultrasonography. Ewes determined to be pregnant were balanced for breed and ram exposure, and randomly assigned to 1 of 3 diets: 100, 60, or 140% National Resource Council (NRC) requirements for total digestible nutrients (TDN) for ewes pregnant with twins [15]. Ewes were fed a complete pelleted feed (Central Connecticut Farmer’s Co-Op, Manchester, CT) which contained 12.8% crude protein, 31.10% acid digestible fiber, 42.10% neutral digestible fiber, and 74.00% total digestible nutrients (TDN). Rations were calculated weekly on an individual BW basis and fed daily to provide 100, 60, or 140% of NRC requirements for TDN for gestating ewes. Ewes were transitioned onto diets at d 31 ± 1.3 of gestation and remained on their respective diets until parturition. Individual housing ensured proper feed intake and any refusals were measured. No refusals were present from any ewe in any group on any day. Upon parturition, lambs from control-fed ewes (CON; n = 12), lambs from over-fed ewes (OVER, n = 12) and lambs from restricted-fed ewes (RES; n = 12) were allowed to nurse their mother for colostrum for up to 24 h. Within 1 d of parturition, one lamb was removed from the ewe for use on study and any remaining lambs and the ewe were returned to the flock. A total of 62 lambs were born to all ewes (Dorset = 44; Southdown = 7; Shropshire = 11). Actual distribution of singletons, twins and triplets were singleton (control-fed = 4; restricted-fed = 0; over-fed = 4); twins (control-fed = 7; restricted-fed = 10; over-fed = 7); triplets (control-fed = 1; restricted-fed = 2; over-fed = 1). The largest of the lambs was chosen when lambs were the same gender. If lambs were different genders, the largest male lamb was chosen for use on the study since males are often slaughtered for market and females kept for breeding. One-half of the lambs from each diet group were slaughtered within 1 d of birth. The remaining lambs were maintained on the same control diet until 3 mo of age regardless of maternal dietary treatment. These lambs were fed milk replacer (1.7% of BW; Land O’Lakes Animal Milk Product Company; Shoreview, MN) from a bottle until weaning at 60 d of age and allowed ad libitum access to water, creep feed (Lamb BT, Blue Seal Feeds; Litchfield, CT), and second cutting hay for the entire 3 mo period. Feed intake was not measured for lambs. Three animals in the 3 mo group died due to causes unrelated to the study. At time of slaughter and sampling, animals were considered fed. The final distribution for gender across treatments was as follows: CON (d 1 = 3 rams, 3 ewes; 3 mo = 3 rams, 3 ewes), RES (d 1 = 3 rams, 3 ewes; 3 mo = 5 rams) and OVER (d 1 = 4 rams, 2 ewes; 3 mo = 3 rams, 3 ewes).

### Sample collection and processing

Animals were euthanized with an intravenous injection of Beuthanasia-D Special (Merck Animal Health; Summit, NJ) containing 390 mg/mL sodium pentobarbital and 50 mg/mL phentoin based on BW, followed by exsanguination. Muscle samples were collected from the midpoint of the left semitendinosus immediately after euthanasia. Samples for RNA or protein extraction were immediately snap frozen in liquid nitrogen. Muscle samples for histology were embedded in Tissue-Tek OCT (Fisher Scientific, Pittsburgh, PA) and frozen in dry ice-cooled isopentane. Samples were stored at −80°C until further use.

### Immunohistochemistry

To determine muscle fiber cross-sectional area (CSA), 10 μm muscle sections were collected using a Leica CM 3050S cryostat (Wetzlar, Germany). Sections were rehydrated in PBS with 0.1% TritonX-100 (PBS-T) for 5 min and fixed with 4% paraformaldehyde (PF) for 10 min. To determine myonuclear number, Hoescht 33342 (1:1,000, Invitrogen, Carlsbad, CA) was used to visualize nuclei and Alexafluor 568 conjugated wheat germ agglutinin (WGA, 1:50, Invitrogen, Carlsbad, CA) was used to visualize the sarcolemma [16]. The number of myonuclei per fiber was determined by dividing the number of myonuclei by the number of muscle fibers in a 20X image. To identify muscle fiber types, cross sections were incubated for 30 min in 5% horse serum in PBS-T to block non-specific antigen sites. Sections were incubated for 1 h with anti-bovine type IIB myosin heavy chain (1:10, BF-F3, Developmental Studies Hybridoma Bank), anti-bovine type IIA myosin heavy chain (1:10, SC-71, Developmental Studies Hybridoma Bank), anti-bovine myosin heavy chain slow (1:10, BAD5, Developmental Studies Hybridoma Bank), and anti-dystrophin (1:50, Pierce, Rockford, IL). Following extensive washing with PBS, sections were incubated with the appropriate AlexaFluor antibodies (1:1,000) and Hoescht 33342 for 30 min. Sections were rinsed with PBS. To quantify intramuscular adiposity, sections were blocked with 5% horse serum in PBS-T for 1 h followed by three 5 min washes with PBS. The sections were incubated with 4,4-Difluoro-1,3,5,7,8-Pentamethyl-4-Bora-3a,4a-Diaza-s-Indacene (BODIPY 493/503, 1:400, Invitrogen D-3922), WGA (1:400), and Hoescht 33342 (1:1,000) in PBS for 1 h in the dark in a humidified box. Following extensive washing with PBS, sections were cover-slipped with 9:1 glycerol/PBS solution. Images for all immunohistochemistry procedures were captured using an AxioCam camera (Zeiss, Jena, Germany) mounted to an AxioObserver microscope (Zeiss) or an Orca ER camera (Hamamatsu, Boston, MA) mounted to an Axiovert Widefield microscope (Zeiss), false colored and merged using ImageJ (NIH). Cross-sectional area was measured as the region within the fiber boundary using the area measurement tool in ImageJ. At least 10 images were obtained from 4 different muscle sections, resulting in the analysis of a minimum of 500 fibers per muscle. Intramuscular adiposity was quantified as a percent of the total area stained in 25 images per animal [17].

### RNA extraction

Tissue was homogenized using the Qiagen Tissuelyser system with 1 mL TriReagent (Sigma Aldrich). A Qiagen Mini Kit was used to extract RNA according to the manufacturer’s protocol (Qiagen; Valencia, CA). Genomic DNA was removed from samples using a Turbo DNA Free kit (Ambion, Foster City, CA). The quality of RNA was determined using a Bioanalyzer analysis system (Agilent Technologies, Santa Clara, CA).

### Real-time reverse transcription (RT)-PCR

Reverse transcription was performed using 300 ng total RNA with OligodT primer (Ambion) and master mix containing 5.5 μL of 5X Buffer (Invitrogen), 1.0 μL dNTP (Promega, Madison, WI), 2.0 μL DTT and 0.5 μL Superscript II (Invitrogen) for a total reaction volume of 20 μL. The samples and master mix underwent a standard RT protocol starting at 70°C for 10 min, 4°C for 20 min, 37°C for 3 min, 42°C for 1 h, 4°C for 3 min, 90°C for 2.5 min. Real-time RT-PCR primers were designed using Primer3 and NCBI BLAST, validated as previously described [18] and synthesized by Integrated DNA Technologies (Coralville, IA). Primer sequences are presented in Table 1. Real-time RT-PCR was performed using Power SybrGreen Master Mix (Invitrogen) and the ABI 7900 HT Fast Real-time PCR machine (Applied Biosystems, Foster City, CA) as previously described [19]. The total volume of the reaction mixture was 25 μL (5 μL cDNA, 3 μL nuclease free water, 1 μL each 10 nmol/L forward and reverse primer, and 10 μL SybrGreen). Real-time RT-PCR was performed using standard cycling conditions (Stage 1: 50°C for 2 min and 95°C for 10 min, Stage 2: 95°C for 15 s and 60°C for 1 min for 40 cycles, Stage 3: 95°C for 15 s and 60°C for 15 s with a 2% ramp to 95°C for 5 min). ^Δ^Ct values were obtained and used to calculate the ^ΔΔ^Ct values to determine relative gene expression [20]. Glyceraldehyde 3-phosphate dehydrogenase (GAPDH) mRNA expression was used as the internal control and did not differ between treatment groups (*P* ≥ 0.25).

**Table 1:** Primer sequences

**Gene**	**Primer sequences (5’ → 3’)**	**Amplicon length**	**Reference**
*Pax7*			
Forward	GAGACCGACTGCTGAAGGAC	167	XM_002685738
Reverse	ATGCTGTGCTTGGCTTTCTT		
*Myf5*			
Forward	AGACGCCTGAAGAAGGTGAA	134	XM_004006219.1
Reverse	AGCAGCTCCTGCAGACTCTC		
*Myogenin*			
Forward	TGGGCGTGTAAGGTGTGTAA	169	Tong et al., 2009 [12]
Reverse	TGCACAGGATCTCCACTTTG		
*MyoD*			
Forward	CCCTGGTGACTTCAGCTGTT	239	Tong et al., 2009 [12]
Reverse	CCTGCCTGCCGTATAAACAT		
*Myostatin*			
Forward	CACAGAAGGTCTTCCCCTCA	147	NM_001001525.2
Reverse	GGTTAAATGCCAACCATTGC		
*Follistatin*			
Forward	AAAACCTACCGCAACGAATG	120	NM_001257093.1
Reverse	GAGCTGCCTGGACAGAAAAC		
*Foxo1*			
Forward	TGACTTGGACGGCATGTTTA	157	XM_004012275.1
Reverse	CCAGCTGTGTGTTGTCGTCT		
*Foxo3a*			
Forward	GGGGAGTTTGGTCAATCAGA	170	NM_001267889.1
Reverse	TTTGCATAGACTGGCTGACG		
*GAPDH*^*1*^			
Forward	GGCGTGAACCACGAGAAGTATAA	118	Buza et al., 2009 [21]
Reverse	CCCTCCACGATGCCAAAGT		

^1^glyceraldehyde 3-phosphate dehydrogenase.

### Western blotting and analyses

Whole muscle protein was extracted by a standard bead beating method (TissueLyser II, Qiagen) in SDS-PAGE sample buffer (250 mmol/L Tris, pH 6.8, 8% SDS, 40% glycerol, and 0.4% β-mercaptoethanol). Protein content was measured by DC Protein Assay (BioRad, Hercules, CA) and an equal amount of protein was separated through 12% polyacrylamide gels. Proteins were transferred to polyvinylidene difluoride (PVDF) membranes and blocked with blocking buffer (Licor Biosciences, Lincoln, NE) for 1 h with gentle agitation. Blots were incubated with primary antibodies overnight at 4°C with agitation. Following extensive washing, blots were incubated with the appropriate infrared secondary antibody. Blots were washed with PBS-T, scanned with the Licor Odyssey Scanning system, and analyzed with Image Studio (Licor Biosciences). After analysis, blots were converted to black and white, inverted, and cropped for presentation (Adobe Photoshop CS5, San Jose, CA). All analysis was done before any adjustment. Antibodies used were: anti-phospho Akt (ser473; 1:1,000, Cell Signaling Technologies, Beverly, MA), anti-phospho Akt (thr308; 1:1,000, Cell Signaling Technologies), anti-Akt (1:1,000, Cell Signaling Technologies), anti-myostatin (N-19; 1:200, Santa Cruz Biotechnology, Dallas, TX), anti-follistatin (H-114; 1:200, Santa Cruz Biotechnology), anti-tubulin (loading control; 0.5 μg/mL, Thermo Scientific, Waltham, MA), goat anti-mouse IRDye 800CW (1:2,000, Licor Biosciences), and goat anti-rabbit IRDye 680RD (1:2,000, Licor Biosciences).

### Statistical analysis

Data were sorted by age (1 d or 3 mo of age) and analyzed using the PROC MIXED procedure (SAS Inst. Inc., Cary, NC) using dietary treatment as the fixed effect. Treatment mean comparisons were performed using LSMEANS statement and PDIFF option. Differences were determined to be significant at *P* ≤ 0.05 or a tendency at 0.05 < *P* ≤ 0.10. Gender and breed differences were not evaluated due to the limited number of females, Shropshire and Southdown lambs in each treatment at each time point. For CSA, fiber type composition, and lipid accumulation, 3 lambs per treatment group were used at d 1 (n = 9) and 3 mo of age (n = 9). For protein expression analysis, 4 lambs per treatment group were used at each time point. For gene expression analysis, 15 lambs were used at 1 d (CON: n = 4, RES: n = 6, and OVER: n = 5) and 12 lambs were used at 3 mo (CON: n = 5, RES: n = 4, and OVER: n = 3).

## Results

By the end of gestation, restricted and overfed diets effectively reduced (−18.9%; 18.3 ± 3.6 kg; *P* < 0.01) or increased (6.6%; 119.7 ± 3.6 kg; *P* < 0.10) ewe body weight (BW), respectively compared with control-fed ewes (112.9 ± 3.6 kg). Overall, OVER lambs weighed 13% more than CON lambs from 1 d to 3 months of age [*P* ≤ 0.05; [22]]. However, there were no differences in BW between RES and CON lambs from 1 d to 3 months of age (*P* = 0.70).

**Figure 1:**
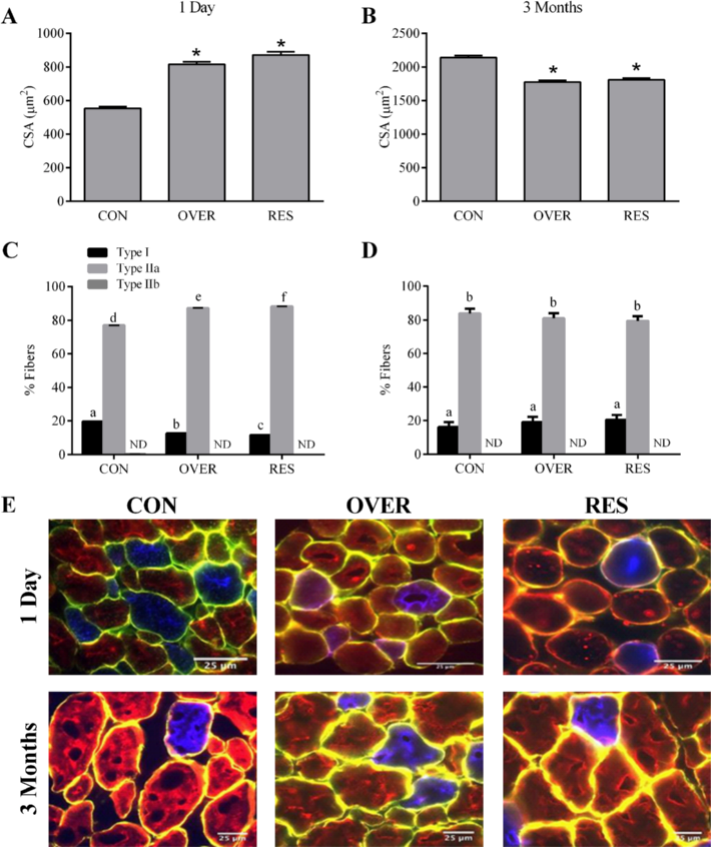
**Poor maternal nutrition alters cross sectional area and fiber type composition of offspring.** Muscle cross sections (10 μm) were immunostained with α-dystrophin to mark muscle fiber membranes (yellow), α-myosin heavy chain (MyHC) I (red), α-MyHC IIA (blue), or α-MyHC IIB (green) to identify the different muscle fiber types. Cross sectional area was measured at 1 d **(A)** and 3 mo **(B)**. Fiber type distribution was determined at 1 d **(C)** and 3 mo **(D)**. Representative photographs are shown **(E)**. *P ≤ 0.05 compared with CON, different letters indicate *P* ≤ 0.05 compared with CON. CON = lambs from control-fed ewes (100% NRC), OVER = lambs from overfed ewes (140% NRC), RES = lambs from restricted-fed ewes (60% NRC), n = 3 lambs/group.

**Figure 2:**
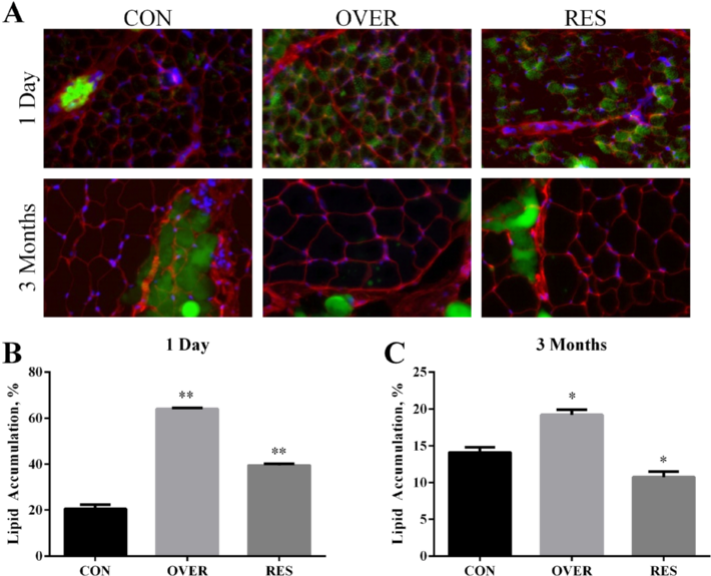
**Poor maternal nutrition alters lipid accumulation in the semitendinosus muscle of offspring.** Cross sections (10 μm) of the semitendinosus muscle were stained with wheat germ agluttinin (WGA; red) to delineate the muscle fiber membrane, BODIPY 493/503 (green) which stains lipids and Hoescht 33342 (blue) to identify nuclei. Representative photographs are shown **(A)**. Data from 1 d **(B)** and 3 mo of age **(C)** are presented as mean ± SEM. **P < 0.0001 vs. CON; *P < 0.05 vs. CON. CON = lambs from control-fed ewes (100% NRC), OVER = lambs from overfed ewes (140% NRC), RES = lambs from restricted-fed ewes (60% NRC), n = 3 lambs/group.

The effects of maternal nutrition during gestation on muscle development were examined in the semitendinosus (**STN**) muscle of a subset of lambs at 1 d and 3 mo of age. At d 1, the CSA of muscle fibers from the STN muscle of OVER and RES lambs was 47% and 57% greater than CON lambs, respectively (*P* < 0.0001, Figure 1A). At 3 mo of age the CSA of OVER and RES lambs was 17% and 15% less, respectively, than the CSA of CON lambs (*P* < 0.0001, Figure 1B). Differences in CSA could have resulted from alterations in myonuclear accretion, thus the number of myonuclei per fiber was calculated. The number of myonuclei per fiber was not different between treatment groups at 1 d (*P* = 0.16) or 3 mo (*P* = 0.32) of age (Table 2). The number of Type IIa fibers was 13.3% greater in OVER lambs and 14.6% greater in RES lambs compared with CON lambs at d 1 (*P* < 0.01, Figure 1C). The percent of Type I fibers was decreased by 35.4% and 40.5% in OVER and RES, respectively, compared with CON (*P* < 0.01, Figure 1C). There was no difference in the muscle fiber composition of the OVER or RES lambs compared with CON lambs at 3 mo of age (*P >* 0.1, Figure 1D). No Type IIb fibers were identified in the STN muscle of any of the lambs. Changes in CSA were not specific to Type I or Type IIa fibers at either age (Table 3). At d 1 lipid content in STN muscle from OVER and RES lambs was 212.4% and 92.5% greater (*P* < 0.0001, Figure 2B) compared with CON lambs. At 3 mo of age, OVER lambs had a 36.1% greater (*P* = 0.003) while RES lambs had 23.6% less (*P* = 0.03) lipid accumulation compared with CON lambs (Figure 2C).

**Table 2:** **Number of myonuclei per muscle fiber**^**1 **^**in the semitendinosus muscle**

**Treatment**^**2**^	**Day 1**	**3 Months**
CON	0.57 ± 0.13	0.72 ± 0.08
OVER	0.37 ± 0.06	0.62 ± 0.13
RES	0.30 ± 0.02	0.46 ± 0.02

^1^Mean ± SEM.

^2^CON = lambs from control-fed ewes (100% NRC), OVER = lambs from overfed ewes (140% NRC), RES = lambs from restricted-fed ewes (60% NRC), n = 3 lambs/group.

**Table 3:** **Cross-sectional area (CSA)**^**1 **^**of Type I and Type IIa fibers in the semitendinosus muscle**

	**Day 1**		**3 Months**	
**Treatment**^**2**^	**Type I, CSA**^**3**^	**Type IIa, CSA**	**Type I, CSA**	**Type IIa, CSA**
CON	306.9 ± 92.5	337.3 ± 42.3	1,791.1 ± 417.2	1,934.3 ± 119.6
OVER	570.7 ± 80.5	570.2 ± 75.0	1,099.6 ± 219.1	1,109.7 ± 20.4
RES	515.2 ± 55.7	622.0 ± 14.7	1,028.2 ± 46.7	1,151.3 ± 37.5

^1^Mean μm^2^ ± SEM.

^2^CON = lambs from control-fed ewes (100% NRC), OVER = lambs from overfed ewes (140% NRC), RES = lambs from restricted-fed ewes (60% NRC), n = 3 lambs/group.

^3^Cross sectional area, mean ± SEM.

The regulation of postnatal muscle growth is complex, involving many different signaling pathways and cellular functions. The lack of postnatal muscle fiber growth in OVER and RES lambs could be due to alterations in satellite cell function or changes in anabolic/catabolic signaling pathways. Over or restricted nutrient consumption during gestation did not alter expression of *Pax7*, *MyoD*, or *myogenin* in whole muscle tissue of lambs at 1 d (*P* ≥ 0.57) or 3 mo (*P* ≥ 0.24; Table 4) of age.

**Table 4:** **Gene expression**^**1 **^**in semitendinosus muscle of CON**^**2**^**, OVER or RES lambs**

	**Day 1**				**3 Months**			
**Gene**	**CON**	**OVER**	**RES**	***P*-value**	**CON**	**OVER**	**RES**	***P*-value**
*Pax7*	1.38 ± 0.67	1.20 ± 0.27	1.86 ± 0.51	0.70	1.14 ± 0.31	1.42 ± 0.24	1.94 ± 0.77	0.46
*MyoD*	2.16 ± 1.36	3.51 ± 0.90	2.70 ± 0.98	0.88	1.11 ± 0.29	1.01 ± 0.01	1.27 ± 0.29	0.83
*Myogenin*	1.53 ± 0.74	2.13 ± 0.74	2.65 ± 0.71	0.57	1.21 ± 0.35	1.24 ± 0.47	2.41 ± 0.66	0.24
*Myostatin*	1.17 ± 0.36	2.94 ± 0.50^**^	1.63 ± 0.51	0.11	1.02 ± 0.10	1.89 ± 0.26	1.51 ± 0.58	0.36
*Follistatin*	1.03 ± 0.16	1.92 ± 0.39^*^	1.73 ± 0.28^**^	0.09	1.10 ± 0.21	1.56 ± 0.21	1.51 ± 0.34	0.36
*Foxo1*	1.12 ± 0.30	0.64 ± 0.29	0.74 ± 0.29	0.37	1.06 ± 0.16	0.91 ± 0.36	0.32 ± 0.07^*^	0.04
*Foxo3a*	1.60 ± 0.73	0.72 ± 0.26	1.12 ± 0.56	0.70	1.09 ± 0.24	1.42 ± 0.43	0.77 ± 0.14	0.21

^1^Relative to CON, mean ± SEM.

^2^CON = lambs from control-fed ewes (100% NRC, d 1, n = 4; 3 mo, n = 5), OVER = lambs from overfed ewes (140% NRC, d 1, n = 5; 3 mo, n = 3), RES = lambs from restricted-fed ewes (60% NRC, d 1, n = 6; 3 mo, n = 4).

*P < 0.05 compared with CON.

**P < 0.01 compared with CON.

**Figure 3:**
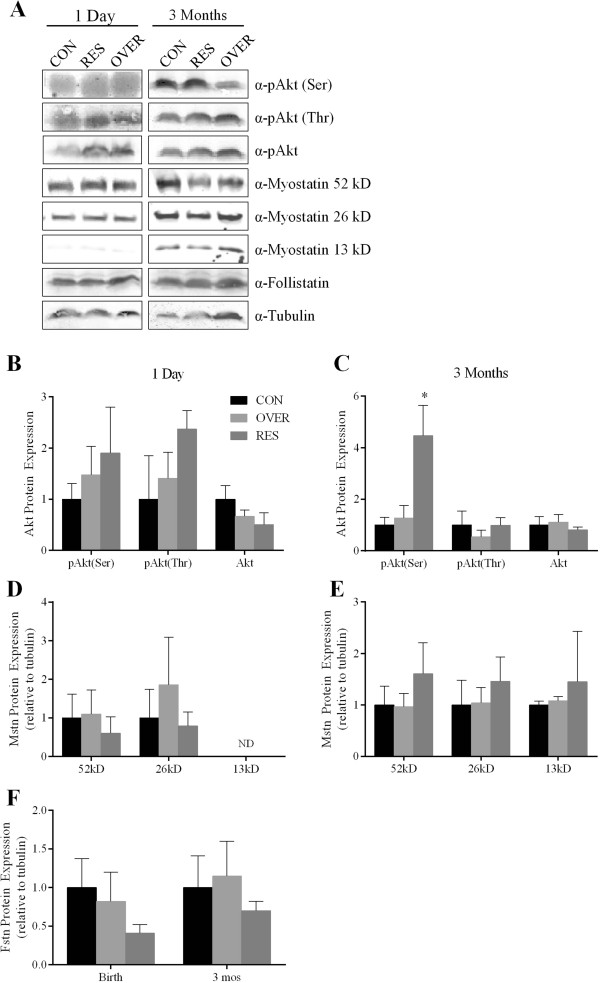
**Restricted maternal nutrition increases Akt phosphorylation postnatally but does not affect myostatin or follistatin protein expression.** Semitendinosus muscle was analyzed for Akt (**B & C**), myostatin (mstn, **D & E**) and follistatin (fstn, **F**) by western blot. Tubulin was used as a loading control. Representative blots are shown **(A)**. Data are presented as mean ± SEM. *P = 0.006. CON = lambs from control-fed ewes (100% NRC), OVER = lambs from overfed ewes (140% NRC), RES = lambs from restricted-fed ewes (60% NRC), n = 4 lambs/group. ND = not detectable.

Reduced CSA at 3 mo of age may be a result of altered expression of myostatin or follistatin, which influence accretion of muscle mass. At d 1, OVER lambs exhibited a tendency toward increased *myostatin* gene expression (*P* = 0.06; Table 4); however no change was observed in RES lambs (*P* = 0.78). At d 1, *follistatin* gene expression was greater in OVER lambs (*P* = 0.04) and tended to be increased in RES lambs (*P* = 0.06, Table 4). No change in *myostatin* or *follistatin* gene expression was observed at the 3 mo time point (*P* = 0.36, Table 4). Further, over or restricted maternal nutrition did not affect the expression of the precursor (*P* = 0.5), active dimeric (*P* = 0.7) or the active monomeric (*P* ≥ 0.7) forms of myostatin protein at d 1 or 3 mo of age (Figure 3D, E). The active monomeric isoform of myostatin was not detectable at d 1. There was also no effect of poor maternal nutrition on the expression of follistatin protein in lambs at 1 d (*P* = 0.5) or 3 mo of age (*P* = 0.7, Figure 3F).

The balance of protein accretion and degradation is crucial to the maintenance of muscle mass and muscle hypertrophy. Increased transcription of forkhead box O1 (*FoxO1*) and *FoxO3a* is associated with catabolic states in muscle [23-26]. At 3 mo of age, reduced *FoxO1* mRNA expression was observed in RES lambs (*P* = 0.04, Table 4). No change was observed in *FoxO1* mRNA expression at d 1 (*P* = 0.37) or *FoxO3a* mRNA expression at d 1 or 3 mo (*P* ≥ 0.20, Table 4). Increased signaling through the insulin receptor substrate/phosphoinositide-3-kinase (IRS/PI3K)-Akt axis is associated with muscle growth [27]. There were no significant effects of poor maternal nutrition on the expression of pAkt (ser473), pAkt (thr308) or total Akt protein at d 1 (*P* ≥ 0.30; Figure 3B). There was no effect of maternal diet composition on the amount of phosphorylated Akt (thr308) or total Akt protein expression at d 1 (*P* ≥ 0.3; Figure 3B) or 3 mo of age (*P* ≥ 0.7; Figure 3C). However, at 3 mo of age, pAkt (ser473) increased 4.5-fold in RES lambs compared with CON (CON: 1.0 ± 0.3; RES: 4.5 ± 1.2; *P* = 0.006; Figure 3C), whereas OVER lambs had similar expression of pAkt (ser473) to CON (*P* = 0.6).

## Discussion

In the present study, over or restricted maternal nutrition during gestation altered muscle growth and lipid accumulation postnatally, muscle fiber type at d 1, postnatal muscle gene expression, and postnatal phosphorylation of Akt in the offspring at 3 mo of age. Thus, poor maternal nutrition during gestation, whether restricted- or over-feeding, impairs muscle development and initiates lasting negative effects on muscle growth of the offspring.

The total number of muscle fibers in mammals is predetermined at birth [28-30]; thus the prenatal development of muscle fibers is crucial for normal muscle growth and function [28,29,31,32]. Importantly, muscle growth is permanently impaired by poor maternal nutrition, both under- and over-feeding [29]. The thrifty phenotype hypothesis suggests that a poor nutrient environment prenatally programs the animal for a poor environment after birth [33]. Given the timing of the poor nutrition (starting at d 31 ± 1.3 d of gestation), it is likely that secondary and tertiary myogenesis were affected resulting in the development of fewer, larger muscle fibers in OVER and RES lambs. This is consistent with other work that suggests that secondary myofibers are preferentially affected by environmental conditions, including nutrition [34]. The poor postnatal muscle growth observed in OVER lambs may be due to prenatally programmed changes in nutrient partitioning, resulting in decreased muscle mass and increased adiposity at 3 mo of age. Although not measured in the current study, changes in fibrosis may also result in decreased muscle mass. Indeed, both maternal obesity and nutrient restriction result in increased collagen content and crosslinking in the muscle [35,36]. However, RES lambs exhibited increased lipid accumulation in the muscle at 1 d of age but decreased lipid content at 3 mo compared with CON lambs, indicating that the mechanisms by which poor maternal nutrition affects both muscle growth and fat deposition in the muscle is specific to the nutritional insult during gestation. In other studies, maternal obesity during gestation increased lipid content in fetal muscle and offspring at 22 mo of age [6,17] whereas nutrient restriction from 28 to 78 d of gestation increased intramuscular triglyceride content of the longissimus muscle in offspring at 8 mo of age [5]. Differences between the current work and that of others may be due to differences in the timing and length of poor nutrition, the severity of over- or under-feeding, or strategies for postnatal feeding. In our study, lambs were removed from the ewe and bottle fed until weaning to remove the impact of changes in milk quantity and composition due to differences in maternal dietary intake during gestation [37]. Importantly, the changes in muscle growth and fat deposition in the current experiment occurred in a flock representative of a production flock, which are heterogeneous for number and gender of the offspring.

Muscle fiber type is related to oxidative capacity [38], insulin sensitivity [39], and meat quality [40]. Alterations in fiber type composition due to poor maternal diet have been conflicting. Under-nutrition during gestation has been shown to increase [5,41] or decrease [42] the number of Type II fibers. Likewise, over-nutrition during gestation decreased [43,44] or had no effect [29] on the percent of Type II fibers. In the current study, the percent of Type II fibers is increased due to restricted- or over-feeding at d 1. The contrasting results are likely due to differences in species, developmental stages, and the timing and/or duration of maternal dietary changes. The availability of an optimal diet postnatally may have allowed for fiber type switching [45] as there was no difference in the fiber type composition of the STN muscle at 3 mo of age. Interestingly, there was no effect of poor maternal nutrition on the CSA of Type I and Type IIa fibers, suggesting that regulation of fiber size by maternal nutrition is not fiber type specific, consistent with previous reports [28,46].

In an effort to understand the factors that may be involved in the phenotypic changes resulting from a poor maternal diet during gestation, we investigated the expression of several factors that regulate muscle development and growth. The lack of fiber growth in OVER or RES lambs could result from failure of a number of mechanisms, including alterations in satellite cell function or changes in anabolic/catabolic mechanisms. Calves from restricted-fed dams possess larger muscle fibers with fewer Pax7 immunopositive cells at d 85 of gestation [36]. The lack of change in *Pax7, MyoD*, and *myogenin* mRNA expression in lambs at d 1 and 3 mo in the current study suggests that changes in satellite cell number and/or function may be more evident in the prenatal animal. The protein kinase Akt has a pivotal role in many cell processes including proliferation, glucose metabolism and angiogenesis [47]. Activated (phosphorylated) Akt enhances skeletal muscle growth through increased protein synthesis and inhibition of protein degradation [27]. Akt is fully activated when phosphorylated at Thr308 and Ser473 [48]. The threonine residue is phosphorylated by pyruvate dehydrogenase kinase (PDK)-1 which leads to phosphorylation of the serine residue. The mechanisms which result in Ser473 phosphorylation are currently under investigation, but may be due to autophosphorylation [48] or phosphorylation by several other candidate kinases, including mTORC2 [49-51]. Constitutively active Akt induces hypertrophy of transfected muscle fibers [52,53] and in muscle-specific transgenic models [27,54]. Further, in response to the activation of IRS/PI3K pathway, Akt phosphorylates FoxO1, blocking its nuclear localization and transcriptional activity [55]. FoxO1 regulates muscle metabolism and protein breakdown and increased mRNA expression is associated with catabolic states in muscle [23-26,56,57]. In this study, Akt phosphorylation at Ser473 was increased and *FoxO1* mRNA expression was reduced in 3-mo old offspring born to restricted-fed mothers, suggesting alterations to the signaling pathways that control protein synthesis/degradation. The upstream pathways controlling increased Akt activity in RES animals at this time point are unclear, but may be related to changes in circulating growth factors. It is unlikely due to changes in circulating insulin like growth factor (IGF) or IGF binding proteins (IGFBP) as there was no difference in the concentrations of these proteins between RES and CON animals [22]. However, the reduced CSA in RES lambs at 3 mo suggests that changes in Akt phosphorylation are not sufficient to overcome the deficit in muscle growth at this time. Indeed, at 8 mo of age, lambs subjected to maternal nutrient restriction during gestation exhibited greater muscle fiber diameter than control animals [5]. Increased signaling through the Akt pathway at 3 mo may provide a mechanism for growth later in life. Interestingly, whereas similar effects were observed in CSA changes in OVER and RES lambs, overfeeding during gestation did not affect activation of Akt or mRNA expression of *FoxO1* in the semitendinosus of lambs at d 1 or 3 mo of age, suggesting that poor postnatal growth in these animals may occur through a separate mechanism.

Myostatin is a member of the transforming growth factor (TGF)-β family which negatively regulates myogenesis at multiple levels [58,59]. Myostatin inhibits muscle growth and development via Smad dependent and independent pathways resulting in decreased protein synthesis and satellite cell proliferation [60,61]. No change in myostatin protein was observed at d 1 or 3 mo in the offspring of poorly nourished ewes. Similarly, no change in follistatin protein, an antagonist of myostatin activity, was observed. However, *myostatin* mRNA expression tended to be increased at d 1 in lambs from poorly nourished ewes, which may be a result of post-translational regulatory mechanisms. Nutritional status can effect microRNA expression resulting in changes in myostatin protein expression in rats and humans [62,63]. In sheep, microRNA can regulate myostatin protein expression [61]. The microRNA regulation of translation may explain the lack of increase in myostatin protein expression observed despite the tendency for an increase in *myostatin* gene expression. Although the regulatory mechanisms are not fully understood, our observations suggest that post-translational modifications may be involved and justify additional studies.

## Conclusions

In summary, over or restricted maternal nutrition in ewes during gestation altered CSA, lipid accumulation, muscle fiber type, and gene and protein expression in the semitendinosus muscle tissue of offspring, resulting in poor postnatal muscle growth. Thus, maternal nutrition during gestation is a critical factor in determining the growth potential of the offspring. The mechanisms by which these changes occur are currently unknown and warrant significant further investigation.

## Competing interests

The authors declare that they have no competing interests.

## Authors’ contributions

SAR – conceived of study, participated in design and coordination, drafted the manuscript. JSR – carried out histochemical analysis and western blots, assisted with statistical analysis. MLH – carried out gene analysis, performed statistical analysis. SAZ – conceived of study, participated in design and coordination, critically evaluated the manuscript. KEG - conceived of study, participated in design and coordination, critically evaluated the manuscript. All authors read and approved the final manuscript.
